# Abdominal Pain in Inflammatory Bowel Disease: An Evidence-Based, Multidisciplinary Review

**DOI:** 10.1093/crocol/otad055

**Published:** 2023-09-26

**Authors:** Matthew D Coates, Kofi Clarke, Emmanuelle Williams, Nimalan Jeganathan, Sanjay Yadav, David Giampetro, Vitaly Gordin, Sadie Smith, Kent Vrana, Anne Bobb, Thu Thi Gazzio, Heather Tressler, Shannon Dalessio

**Affiliations:** Department of Medicine, Division of Gastroenterology & Hepatology, Penn State College of Medicine, Hershey, PA, USA; Department of Pharmacology, Penn State College of Medicine, Hershey, PA, USA; Department of Medicine, Division of Gastroenterology & Hepatology, Penn State College of Medicine, Hershey, PA, USA; Department of Medicine, Division of Gastroenterology & Hepatology, Penn State College of Medicine, Hershey, PA, USA; Department of Surgery, Division of Colorectal Surgery, Penn State College of Medicine, Hershey, PA, USA; Department of Psychiatry, Penn State College of Medicine, Hershey, PA, USA; Department of Anesthesia & Perioperative Medicine, Penn State College of Medicine, Hershey, PA, USA; Department of Anesthesia & Perioperative Medicine, Penn State College of Medicine, Hershey, PA, USA; Department of Anesthesia & Perioperative Medicine, Penn State College of Medicine, Hershey, PA, USA; Department of Pharmacology, Penn State College of Medicine, Hershey, PA, USA; Department of Surgery, Division of Colorectal Surgery, Penn State College of Medicine, Hershey, PA, USA; Department of Surgery, Division of Colorectal Surgery, Penn State College of Medicine, Hershey, PA, USA; Department of Medicine, Division of Gastroenterology & Hepatology, Penn State College of Medicine, Hershey, PA, USA; Department of Medicine, Division of Gastroenterology & Hepatology, Penn State College of Medicine, Hershey, PA, USA

**Keywords:** abdominal pain, inflammatory bowel disease, Crohn’s disease, ulcerative colitis

## Abstract

Abdominal pain is one of the most common and impactful symptoms associated with inflammatory bowel disease (IBD), including both Crohn’s disease and ulcerative colitis. A great deal of research has been undertaken over the past several years to improve our understanding and to optimize management of this issue. Unfortunately, there is still significant confusion about the underlying pathophysiology of abdominal pain in these conditions and the evidence underlying treatment options in this context. There is also a relative paucity of comprehensive reviews on this topic, including those that simultaneously evaluate pharmacological and nonpharmacological therapeutic options. In this review, our multidisciplinary team examines evidence for various currently available medical, surgical, and other analgesic options to manage abdominal pain in IBD.

## Graphical Abstract

**Graphical Abstract:**
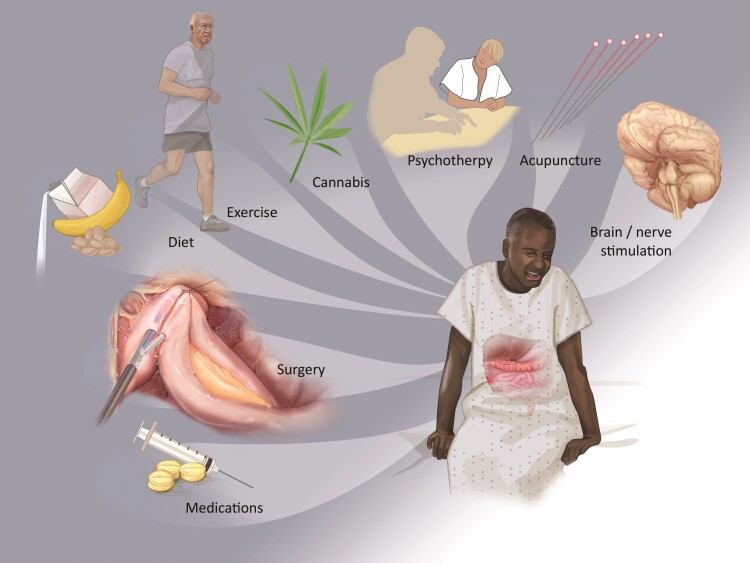

## Introduction

Inflammatory bowel disease (IBD) encompasses a set of disorders, including Crohn’s disease (CD) and ulcerative colitis (UC), that are characterized by chronic relapsing inflammation of the gastrointestinal tract. IBD is estimated to affect over 3 million individuals in the United States alone. These are complex disorders that are frequently difficult to manage, in part because of a myriad of problematic symptoms they are associated with. IBD incurs billions of dollars each year in healthcare expenditures and lost work hours.^1,2^ Abdominal pain is one of the most common and important symptoms associated with IBD. It is described by at least 70% of patients experiencing the initial onset or an exacerbation of the disease.^3^ Abdominal pain negatively impacts quality of life in the setting of IBD, and is a major reason patients seek out medical assistance and incur healthcare costs.^4–6^ Considering its outsized importance in determining patient (and caregiver) perception of disease, it is critical for IBD providers to develop a clear understanding of the causes, impacts, and management options for abdominal pain in this setting. Additionally, while many important conceptual inroads have been made about abdominal pain in general, a variety of questions remain regarding the underlying pathophysiology of this symptom in IBD. Thus, there is a great opportunity to make significant strides in our understanding of this issue. In this review, we discuss what is known about abdominal pain in adult IBD based on studies specifically evaluating these particular disorders. We also review current gaps in our knowledge about this topic and questions that need to be addressed in order to improve our ability to optimize pain management strategies in this setting.

## Epidemiology and Impact of Abdominal Pain in IBD

Over 70% of IBD patients report abdominal pain at some point during the course of their disease.^7^ It is frequently described in both UC and CD.^8,9^ Some reports suggest that it is more commonly described in CD than UC^8^ but others suggest that abdominal pain is experienced at comparable rates in these subtypes of IBD.^7^ Women are more likely to experience abdominal pain with IBD than men.^8,9^ Similar to the adult IBD population, abdominal pain is also common in the pediatric IBD population, with over 50% of youth reporting abdominal pain.^10,11^

Abdominal pain is a major driver of cost and healthcare resource utilization (HRU) in digestive disease, including IBD.^12^ CD and UC have both been associated with increased HRU compared to the general population^4,5^ and the associated costs of this care have steadily increased in recent years.^6^ Avoidance of abdominal pain has been described as one of the significant determinants of patient treatment preference.^13^ Importantly, the presence of abdominal pain in IBD, even in the absence of active disease, has also been independently associated with increased HRU,^14^ including increased risk of clinic and hospital visitation, use of IBD-associated medication, and IBD-associated surgery.^15^ It has also been associated with the use of potentially detrimental analgesic agents that can cause their own adverse effects,^16,17^ including nonsteroidal anti-inflammatory drugs (NSAIDs), which can directly damage the gastrointestinal tract, and opioids, which come with a risk of addiction, and can actually exacerbate gastrointestinal symptoms.^17^ Additionally, the use of other common (and seemingly benign) treatments for abdominal pain in IBD (eg, tricyclic antidepressants [TCAs]) has also been associated with other adverse issues, including suicidal ideation.^18^

While the direct medical costs of abdominal pain in IBD are substantial, there are also a variety of indirect costs incurred by this symptom, including missed employment and lost work hours,^19^ as well as reduced levels of physical activity,^20^ and increased risk of disability.^21,22^ Abdominal pain in IBD is also independently associated with significant impacts on health in IBD. It has been linked to weight loss and poor nutritional markers in IBD patients.^23^ Mental well-being is frequently affected, including increased risk of stress, depression, and anxiety,^23,24^ as well as an increased risk of anhedonia.^25^ This is particularly relevant as chronic stress has been associated with disease exacerbations and poor outcomes in IBD and other disorders associated with visceral pain, such as irritable bowel syndrome (IBS).^26,27^ Behaviors such as catastrophizing, poor coping, and fear-based avoidance are also frequently present in IBD patients with abdominal pain.^28^ These debilitating behaviors can result in an increased perception of pain, further causing disability and decreased social functioning.^29,30^ IBD patients with pain are also at increased risk of using substances of abuse, including alcohol, tobacco, opioids, and cannabis.^31,32^

Considering these factors, it is perhaps unsurprising that multiple studies have demonstrated a detrimental impact of abdominal pain on patient perception of disease control and quality of life in IBD.^9,14,17,25,33,34^ In brief, when IBD patients are experiencing abdominal pain, even if their disease process otherwise appears to be under control, they have a relatively negative impression of their health and its management.

## Causes of and Contributors to Abdominal Pain in IBD

**Figure 1.**
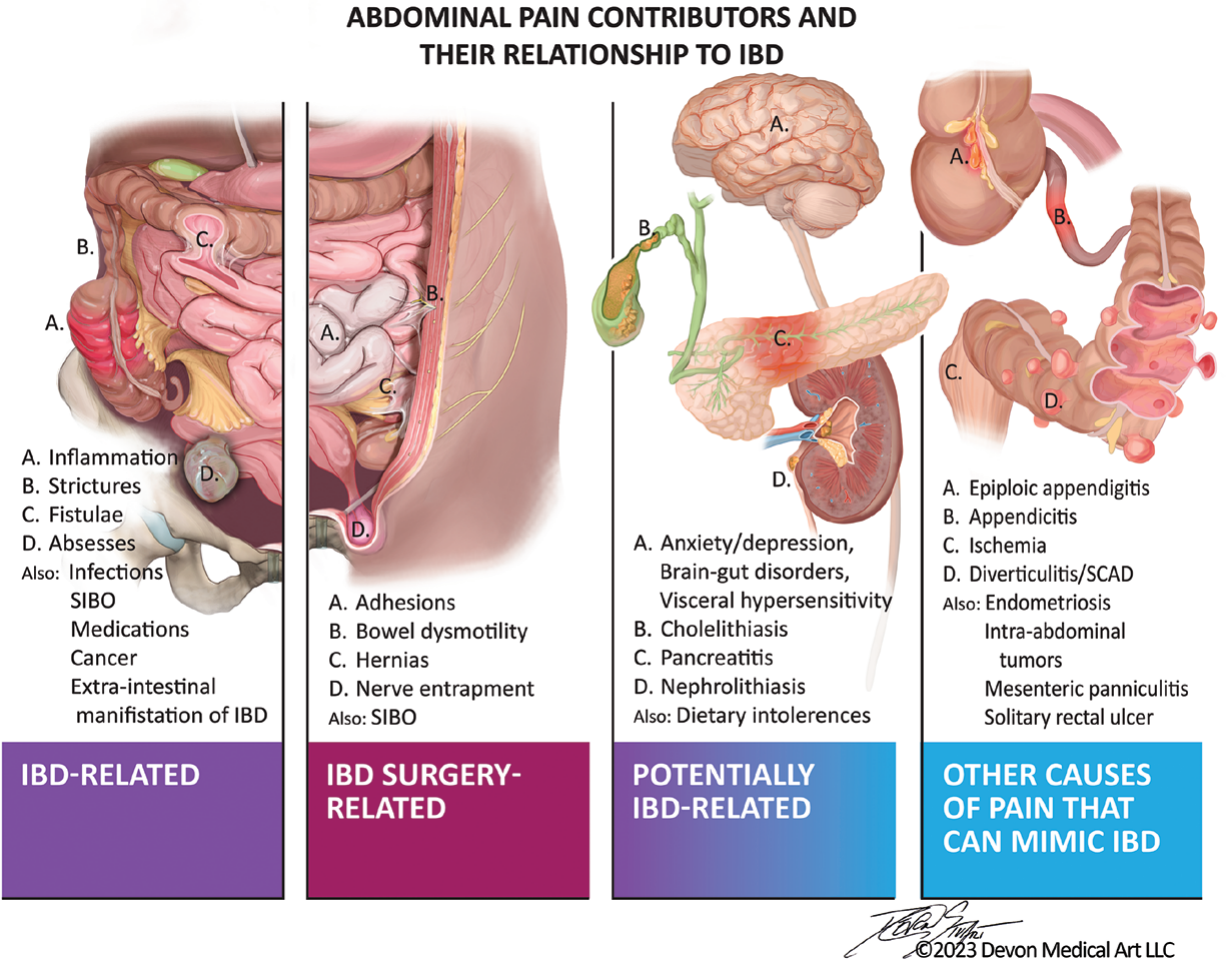
Causes and contributors to abdominal pain in inflammatory bowel disease.

There are a large number and variety of factors that stimulate or influence abdominal pain experience in IBD (Figure 1). The inflammation associated with IBD is considered to be the primary driver of pain, as cytokines and other inflammatory mediators sensitize extrinsic and intrinsic primary afferent neurons found in the gut.^3^ Some evidence suggests that even when patients are considered to be in remission, if they continue to have symptoms including abdominal pain, they still frequently exhibit persistent low-grade inflammation.^35^ In addition to the impacts of the primary inflammatory aspect of IBD, other disease-related complications can cause pain, including gastrointestinal stenoses/strictures, abscesses, and fistulae. The inflammation and these complications may in turn lead to other problems, including infections, bacterial overgrowth, and even cancer, each of which can drive abdominal pain as well.^36–38^ Furthermore, complications of IBD-associated surgeries, such as the development of adhesions and disruptions of bowel motility (through associated obstructive-type phenomena and/or small bowel bacterial overgrowth), hernias, and/or nerve entrapment within the abdominal wall, can also lead to abdominal pain.^39–43^ There are also extraintestinal manifestations of IBD that result in abdominal pain, including cholelithiasis, nephrolithiasis, and pancreatitis.^44–46^

Importantly, many patients with quiescent IBD continue to exhibit persistent abdominal pain that cannot be explained by the presence of active inflammation, its anatomical complications, surgical complications, or other inflammatory or neoplastic conditions.^9,24^ Previous estimates suggest that up to 33% of individuals with UC, and more than 60% of individuals with CD who are in clinical remission, continue to experience persistent symptoms, including chronic abdominal pain.^9,17,24,47,48^ Several other factors can play a role in this setting. This includes the possibility of coexistent functional bowel disorders, such as IBS. Functional bowel disorders (also known as brain–gut disorders) are exceedingly common, affecting one-quarter or more of the adult population.^49^ These conditions are frequently associated with abdominal pain, and have been reported to be at least twice as likely to occur in IBD patients.^50^ Several psychiatric conditions that influence patient pain experience are more frequently found in IBD than in the general population. For example, symptoms of anxiety, depression, and stress, which can heighten perception of pain, are very common in both CD and UC.^9,24,51^ Somatization and somatoform disorders have also been described and are important to consider as they may lead to abdominal pain perception in the setting of IBD even when an injury or active disease does not exist.^3,52^

Certain medications can also increase the risk of developing abdominal pain in IBD. These include use of NSAIDs such as aspirin, ibuprofen, and naproxen. These agents are known to induce damage in the gastrointestinal mucosa that may mimic or even exacerbate the inflammatory process associated with CD and UC.^53,54^ Ironically, opioid medications also can lead to abdominal pain.^55^ In addition to experiencing an array of detrimental impacts on gut motility that may lead to abdominal discomfort, chronic opioid users are at risk of developing derangements in visceral pain perception.^56^ This includes a condition known as narcotic bowel syndrome, which is characterized by the development of persistent or recurrent abdominal pain that actually worsens with increasing doses of opioids.^55^ In fact, an array of medications has previously been associated with abdominal pain in IBD. A more complete discussion of this topic can be found in the following section describing pharmacological approaches to managing IBD-associated pain.

There is evidence that some foods may increase the chance of experiencing abdominal pain in IBD. These include the fermentable oligosaccharides, disaccharides, monosaccharides, and polyols (FODMAPs), whose consumption has been associated with an increased risk of IBS-like symptoms.^57^ IBD patients have also demonstrated increased risk for demonstrating certain types of food intolerance, including for lactose-containing foods.^58^ These findings are important, in part, because abdominal pain alone (even in the absence of active disease) has been demonstrated to have a significant impact on dietary patterns of IBD patients.^23^

Another possible explanation for abdominal pain in IBD is that patients may have inherent or acquired differences associated with the peripheral and/or central nervous signaling systems responsible for abdominal pain perception. For example, some neuroendocrine signaling systems associated with the gut that are capable of influencing pain experience have been shown to be altered in IBD, including elements of mucosal serotonin signaling.^59,60^ Other studies suggest that nociceptive neurons associated with the gut (ie, those neurons responsible for sensing noxious stimuli associated with pain and transmitting information back to the central nervous system) exhibit alterations in quiescent IBD patients with persistent pain, including increases in the density of transient receptor potential vanilloid type 1 (TRPV1), a key receptor involved in activation of these neurons.^61^ Brain imaging studies have also suggested that IBD (CD) patients with chronic abdominal pain exhibit differential expression of certain neurotransmitters, including glutamate and GABA, when compared to healthy controls and CD patients without pain.^62,63^ There is evidence that these and other signaling changes are associated with variation in gray matter volume as well.^62,64^ Notably, no currently available scientific literature has determined whether any of these changes are modifiable.

Finally, patients can manifest acute or chronic pain-inducing inflammatory phenomena within the abdomen that may not have any relationship to their IBD, including appendicitis,^65,66^ diverticulitis,^67^ segmental colitis associated with diverticular disease (SCAD),^68^ endometriosis,^69^ mesenteric ischemia or ischemic colitis,^70,71^ mesenteric panniculitis,^72^ solitary rectal ulcer,^73^ and epiploic appendigitis.^74^ They can also develop benign and malignant growths (that may or may not relate to IBD and/or the associated medical therapy), including gastrointestinal, ovarian, uterine, testicular, hepatic, renal, and other intra-abdominal tumors that can result in abdominal pain.^75–77^

## Characterizing Abdominal Pain in IBD

Several aspects of a patient’s abdominal pain experience are important to consider when caring for and/or studying this phenomenon. Specifically, if a patient has abdominal pain, it is critical to get a clear sense of its chronicity, frequency, intensity, and character as well as the associated ameliorating and exacerbating factors. Each of these factors provides important clues about the potential etiology. In most cases, all of this can be determined by performing a careful history with the patient.

A variety of more formalized approaches can also be taken to gather this information. To date, no surveys or tools have been validated to specifically assess abdominal pain in IBD patients. However, there are surveys that have been validated to evaluate disease severity in IBD that include measures of abdominal pain severity. Prominent examples include the Crohn’s Disease Activity Index (CDAI)^78,79^ and Short Colitis Clinical Index (SCCAI),^80^ each of which uses a 4-point Likert-type scale of pain intensity. Additionally, surveys validated to measure quality of life, including the Inflammatory Bowel Disease Questionnaire (IBDQ) and Short Inflammatory Bowel Disease Questionnaire (SIBDQ), also measure pain intensity (using a 7-point Likert-type scale).^81,82^ Each of the pain assessment tools utilized by these particular surveys is relatively easy to use but is limited in regard to how well the pain is temporally and qualitatively characterized.

There are other survey tools that have been widely utilized in clinical and research settings that also have significant potential to be helpful in this regard. This includes the Wong–Baker FACES Pain Rating Scale, a simple visual measure of current pain intensity routinely utilized to assess all modalities of pain, including that associated with the abdomen.^83,84^ The McGill Pain Questionnaire and the short-form McGill Pain Questionnaire are lengthier pain surveys frequently employed in research studies that include assessments of pain severity (using another visual analog scale), as well as the qualitative descriptors for affective and sensory aspects of pain.^85,86^

Additionally, there have been attempts to utilize more objective assessments of visceral pain in IBD and other digestive disease disorders. The most common methodology relied upon thus far has been assessment of patient response to barostatic rectal distension.^87^ While this technique has been studied in the setting of IBD, it has been used relatively sparingly as it is difficult to employ in the clinical setting, particularly over repeated time points.^87–89^ As a result, it has also not been specifically validated for clinical use to assess pain in IBD.

## Investigating the Cause(s) of Abdominal Pain in IBD

Recognizing and understanding the source(s) of abdominal pain is essential to managing this symptom, particularly given the myriad forms of abdominal pain described by this patient population. As the inflammation associated with IBD has previously been shown to be the most common driver or contributor to abdominal pain,^3,9,17^ it is essential to get an accurate assessment of current and recent patient health and IBD activity status, in addition to evaluating for the presence of consequential IBD-associated complications, including strictures, fistulae, and abscesses. The first and most fundamental step in this assessment is to take a careful history.^90^ Provider-based evaluations of patient status, such as the physician global assessment, provide subjective information about a patient’s overall status but are based on variable (sometimes poorly described) factors and not particularly objective in nature. Providers and investigators can take a more regimented approach through the use of 1 or more validated surveys, including the CDAI and Harvey–Bradshaw Index (HBI) for CD,^78,79^ and the Truelove & Witt’s Severity Index, Clinical Activity Index (CAI), and SCCAI for UC.^80,91^

As previously demonstrated, though, history-based patient assessments frequently fail to identify individuals with active inflammation.^92,93^ Thus, it is standard practice to pair these approaches with objective measures of IBD activity. Such assessments include serological measures of inflammatory activity (eg, C-reactive protein, sedimentation rate), stool tests (eg, fecal calprotectin), radiological imaging (eg, small bowel follow-through, ultrasound, computed tomography scan, magnetic resonance imaging), and endoscopic evaluation (eg, esophagogastroduodenoscopy, colonoscopy, capsule enteroscopy).^94–96^ Direct endoscopic evaluation of the gastrointestinal tract with mucosal biopsy (using an ileocolonoscopy with or without an upper endoscopic exam) has long been the preferred method for diagnosing and monitoring IBD,^96–98^ but there is evidence that even these tests can miss a significant number of active cases, particularly when they involve deep small bowel CD.^99^ In truth, no single diagnostic approach is completely reliable to rule out active inflammation and so a multifocal testing strategy, utilizing a mixture of historical, serological, radiological, and endoscopic data, is still the most effective way to assess IBD activity.

If abdominal pain exists in the absence of identifiable IBD-associated inflammation, as indicated above, there are a variety of other potential contributors that can be explored. Again, a careful review of the patient’s history is imperative under these circumstances. Patient medication lists should be scrutinized to evaluate for potential offending pharmacological agents. A brief psychiatric assessment by the IBD provider should be performed, and a more formal evaluation by a psychiatrist should be considered. Assessment of a patient’s dietary practices, particularly in the context of their symptomatic experience, can be used to evaluate potential food-related contributors. This includes pain-inducing complications related to IBD, such as strictures, fistulae (both intra-abdominal and perianal), and abscesses. Radiologic and endoscopic assessments can be critical to identify each of these problems.

## Treating Abdominal Pain in IBD

### Optimizing IBD Medical Therapy

The key to effectively managing abdominal pain in IBD is to optimally clarify the source or sources of this symptom. If there is evidence for persistent disease activity, the first and most important step is to treat this using appropriate anti-inflammatory and/or immune-modifying strategies. It is imperative to control the associated inflammatory process, as even milder forms of luminal inflammation may be associated with abdominal pain in IBD.^9,17,24^ Generally speaking, any therapy that has the capability of positively influencing inflammatory activity in IBD also has the potential to positively impact abdominal pain in these conditions. Further discussion regarding the potential for IBD-directed medications to influence abdominal pain experience can be found below.

### Surgery and Abdominal Pain in IBD

Other IBD-associated complications (including luminal strictures, intra-abdominal fistulae, abscesses, and/or even neoplasms) may drive abdominal pain in addition to or independent of gut-based inflammation. In such cases, it may be necessary to consider surgical interventions to properly address these issues. The diagnostic approaches described above are critical for properly monitoring IBD patients describing abdominal pain for these issues and to help optimize their medical and surgical interventions as necessary.

While surgery can be an essential component of the long-term management of abdominal pain in IBD, it also has the potential to cause this symptom. Patients who have undergone abdominal surgery, particularly laparotomy, can develop a variety of complications that can lead to chronic myofascial abdominal wall pain.^100–102^ Patients undergoing abdominal procedures can also develop abdominal pain related to incisional and/or parastomal hernias. Chronic abdominal wall pain may develop as a result of neuromuscular aggravation within the wall of the hernia. Visceral pain may result from impingement or incarceration of associated bowel and/or protrusion of intra-abdominal contents into the hernia itself and, if severe and/or persistent, may require surgical repair to definitively resolve the symptom.^100,103^ Finally, surgical patients can also develop entrapped nerves or neuromas within the abdominal wall at or near healed incisional sites that can cause chronic, severe, localized pain, often made worse with movement of the affected muscles.^104^ In these cases, empiric and/or ultrasound-guided injections at the affected site (“trigger point”), and in the surrounding musculature and associated soft tissue, can be diagnostically and therapeutically beneficial.^101,102^ Regional nerve blocks (including rectus sheath, transabdominis plane, and ilioinguinal/iliohypogastric nerve blocks) have also demonstrated efficacy for management of chronic abdominal wall pain.^105^ With or without surgery, patients can also develop abdominal wall muscle strains. These are usually self-limited injuries that will resolve within days or weeks.^104^ For most patients, these strains can be effectively addressed using supportive therapy, including a combination of rest, heating pads, and/or core musculature strengthening exercises with or without physical therapy.^100,104^ In patients with persistent myofascial abdominal wall pain, though, injections of an anesthetic (eg, bupivacaine, lidocaine) at a trigger point, scar neuroma, and/or the surrounding soft tissue may provide temporary relief (in conjunction with the conservative therapies described above) and can improve participation in physical therapy.

### Nonpharmacological Treatment Options

**Figure 2.**
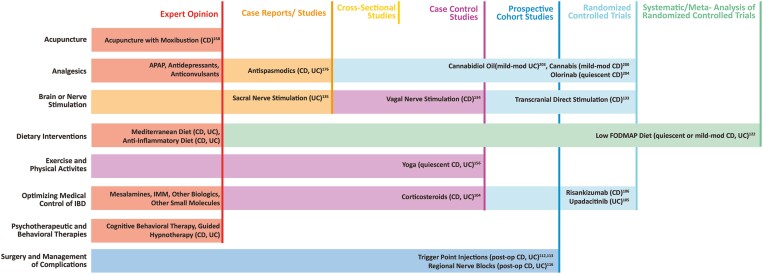
Study types supporting the use of selected treatments for abdominal pain in inflammatory bowel disease.

In the absence of IBD-associated inflammation and/or complications, as previously discussed, patients may still report acute or chronic abdominal pain. This can be debilitating and may exert a devastating impact on patient perception of disease control and quality of life. In these circumstances, there are a wide variety of approaches that can be taken, including the use of both pharmacological and nonpharmacological interventions. Notably, to date, most of the treatment strategies that have demonstrated scientifically demonstrable efficacy in managing abdominal pain in IBD are nonpharmacological options^106,107^ (Figure 2). Here we review the current evidence for their use in IBD.

#### Diet

Several recent studies suggest that monitoring and modifying nutritional intake may be effective for addressing abdominal pain in IBD. While many dietary components have been associated with IBD-symptom aggravation,^108^ few well-defined nutritional approaches have been specifically studied to assess their effects on abdominal pain experience in IBD. To date, the best-studied dietary approach in this context has been the use of the low-FODMAP diet. These constituents are presumed to promote abdominal pain through the production of gas and diarrhea. Halmos and colleagues performed a randomized controlled trial (RCT) that demonstrated that CD patients adhering to a low-FODMAP diet for 21 days reported fewer gastrointestinal symptoms.^109^ Pedersen et al. also undertook an RCT that demonstrated that IBD patients who followed a low-FODMAP diet exhibited reduced IBS-like symptoms compared to those that did not follow this approach.^57^ A separate “single-blind trial” of IBD patients undertaken in the United Kingdom demonstrated that a low-FODMAP diet resulted in a higher proportion of individuals reporting adequate relief of their symptoms, including abdominal discomfort.^110^ Zhan and colleagues performed a meta-analysis, evaluating 6 studies (including the 2 RCTs described above and 4 other studies) investigating the impact of a low-FODMAP diet and found that it was associated with reduced likelihood of abdominal pain along with other problematic IBD-related symptoms (such as diarrhea and bloating).^111^

Modification of dietary fiber type and content has also been evaluated in this context. Generally speaking, in the absence of known severe fibrostenotic complications, consumption of fiber-rich foods is usually recommended in IBD.^112^ Indeed, some investigators have found that higher fiber intake is associated with better outcomes in CD and UC, including reduced abdominal pain.^113,114^ However, other patient-reported data suggest that a high-fiber diet may actually be associated with an increased risk of abdominal pain and other gastrointestinal symptoms.^108^ Considering the complexity of even defining the different forms of dietary fiber and their disparate relative impacts on human gastrointestinal physiology,^115^ it is not surprising that this topic has become an area of controversy. Similarly, even when considering the related common recommendation of maintaining a low “residue” diet in the setting of significant CD-related stricturing, the existing evidence for influence on pain control in this setting is actually very limited and controversial.^116^ To date, no single definition to clarify what constitutes dietary “residue” has been agreed upon.^116^ Thus, the utility of fiber and “residue” modification in this context is still up for debate.

Other diets have been studied in the context of IBD, in part to investigate their potential to impact IBD-related symptoms, including abdominal pain. However, the evidence for their effect on abdominal pain is relatively limited. Use of the Mediterranean diet (MD), which includes high intake of vegetables, fruits, cereals, legumes, unsaturated fat and reduced intake of saturated fats, meat, and sweets, has been associated with improvements in inflammatory markers in IBD in at least 1 prior study.^117^ However, no clear data specifically evaluating the impact of this diet on abdominal pain are currently available. The Specific Carbohydrate Diet (SCD), which focuses primarily on the consumption of monosaccharides, solid proteins, certain vegetables, fruits, and nuts, has been associated with diminished gastrointestinal symptom scores in at least 1 case series,^118^ but its impact on abdominal pain has not been rigorously assessed. Of note, a recent trial comparing use of the MD and SCD in a cohort of CD patients found no significant difference in inflammatory or symptomatic outcomes between these diets in this setting.^119^ The so-called anti-inflammatory diet (AID), an approach that includes consumption of probiotics and prebiotics and limits intake of carbohydrates with modified fatty acids, has demonstrated variable efficacy in addressing the activity and symptoms associated with IBD.^120^ Thus far, no clear determination can be made as to the utility of this approach for managing abdominal pain, as some studies demonstrate improvement in IBD-associated symptoms in the context of AID, while others suggest that symptoms actually worsen.^120^ Additionally, gluten-free and Paleolithic diets have been studied in this context,^121,122^ but the findings have been limited, conflicting, and/or difficult to interpret thus far.

#### Brain and/or nerve stimulation

Techniques designed to directly influence brain activity, including transcranial direct current stimulation (tDCS), have demonstrated potential for management of abdominal pain in a variety of conditions.^123^ In 1 RCT, use of tDCS for 5 days was associated with a significant reduction in abdominal pain severity in a mixed cohort of CD and UC patients.^124^

Limited studies investigating the impact of nerve stimulation on IBD activity and abdominal pain have also been undertaken. For example, in a small (*n* = 7) pilot study, vagal nerve stimulation (VNS) has been evaluated in the setting of CD. All of the patients who underwent VNS for 6 months reported improvement in “perceived digestive pain” scores.^125^ A case study involving a patient with refractory ulcerative proctitis demonstrated improvement in inflammation and symptoms (including abdominal pain) was reported after 3 weeks of treatment with a sacral nerve stimulation device.^126^ The small sample sizes, variable study designs, and outcome measurements make these findings difficult to interpret, however.

#### Psychotherapeutic & behavioral therapies

Psychotherapeutic strategies have also been utilized in this setting. Cognitive behavioral therapy (CBT) is a widely used form of psychotherapy designed to address negative thoughts and behaviors that can adversely impact quality of life and disease symptom experience. Importantly, CBT has seen widespread use in the context of chronic pain^127^ and there is evidence that it can help to improve abdominal pain and other symptoms in functional bowel disorders such as IBS.^128–130^ In IBD, there is evidence that CBT may improve quality of life, in particular in cohorts of patients exhibiting increased stress^131,132^ and/or mood disturbances.^133^ To date, though, despite repeated studies over the past several decades, there is no definitive evidence that CBT significantly influences disease activity or physical symptoms (including abdominal pain) in IBD,^133,134^ including in a 2-year RCT.^132^

Hypnotherapy has also been used to help address pain and other symptoms in a variety of diseases. Gut-directed hypnotherapy (GDH) targets posthypnotic suggestions on gastrointestinal health and symptom management.^134,135^ Previous studies have associated GDH with improvements in quality of life and reductions in gut-based inflammatory mediators in UC,^135^ and a small prospective study demonstrated that UC patients receiving long-term GDH were more likely to achieve clinical remission than those who did not receive GDH.^136^ Several previous investigations have demonstrated that GDH may improve abdominal pain in IBS.^134^ However, studies specifically evaluating the impact of GDH on abdominal pain and/or related symptoms in IBD have not demonstrated a significant impact of this intervention.^135,137^

Mindfulness-based CBT (MBCBT) is another form of psychotherapy designed to help manage symptoms related to stress and depression. Early studies suggested that “relaxation training” could diminish generic pain (though not abdomen-specific) scores in UC patients.^138^ Another small study evaluating the influence of a relaxation-based mind–body group intervention demonstrated relative improvements in pain catastrophizing as a result of this intervention.^139^ Other studies also suggested that use of MBCBT is associated with improved quality of life and reduced perceived stress, depressive and anxious trait scores in IBD.^140,141^ Additionally, there is evidence that use of MBCBT in coordination with standard medical therapies can result in relative improvement of inflammatory markers.^142^ However, a recent meta-analysis investigating MBCBT in IBD demonstrated no significant effect on “physical symptoms” of IBD, including abdominal pain.^143^ Notably, to date, no studies have been undertaken to specifically evaluate the influence of MBCBT on abdominal pain in IBD.

Other behavioral modification factors, including alternative stress management programs, biofeedback, meditation, and optimization of sleep hygiene, have theoretical promise for improving symptom-related outcomes in IBD. However, these techniques have not yet been rigorously evaluated specifically for their utility in managing abdominal pain in this setting.

#### Exercise and physical activities

There is evidence that exercise programs (ie, planned, structured physical activity designed to improve fitness) in IBD are associated with a wide variety of positive outcomes, including measures of disease activity in IBD and reduction in severity or frequency of at least some symptoms (eg, fatigue), particularly in the setting of quiescent disease.^144,145^ How exercise impacts abdominal pain in this population is currently unclear, though, as no study has been designed to carefully evaluate the impact of exercise on this symptom. Studies on exercise in IBD that have included some assessment of abdominal pain have been challenging to interpret due to methodological variability and/or contradictory findings. For example, a survey of 918 IBD patients undertaken in the United Kingdom found that 607 (66%) individuals engaged in some form of exercise on at least a weekly basis. Of the patients that exercised, 72% reported feeling generally better (including 12% who reported improvement in 1 or more IBD-associated symptoms) as a result of the exercise. However, 23% of respondents reported that exercise made them feel worse, including 17% who described worsening abdominal pain.^146^

The impact of yoga on abdominal pain in IBD has also been evaluated. Specifically, 1 prospective study of 100 IBD patients (40 CD, 60 UC) deemed to be in clinical remission (on standard medical therapy) demonstrated that individuals who participated in at least 1 hour of yoga daily over an 8-week span reported a reduced frequency of several symptoms, including “intestinal colic pain.”^147^

Physical therapy has the potential to help with at least certain forms of abdominal pain in IBD, particularly in those associated with abdominal wall issues, such as surgery-related pain, muscle strains, and entrapped nerves.^3,104^ We are aware of no dedicated studies investigating this topic in IBD, however. Thus, no scientific evaluation of the impact of physical therapy has been undertaken yet.

#### Acupuncture

There is significant interest in the application of acupuncture for IBD patients, including as a potential analgesic.^148^ Several smaller-scale studies, utilizing variable approaches, have been undertaken to evaluate acupuncture in IBD. For example, a recent small clinical trial in CD patients performed in China demonstrated a significant positive impact on clinical remission rates (based upon the CDAI) in patients receiving a single treatment of acupuncture with moxibustion (ie, burning dried mugwort on parts of the body) in addition to their baseline IBD-related medical therapies.^149^ Attempts to summarize the totality of findings (including through meta-analyses) have been restricted by limitations on individual study quality and findings as well as consistency in methodological approach.^150,151^

### Pharmacological Treatment Options

As previously detailed in 2 recent Cochrane Reviews, no analgesic medication has demonstrated proven efficacy in improving abdominal pain in IBD.^106,107^ As a result, no medications have been specifically approved to treat abdominal pain in the setting of IBD in the United States. Regardless, there are many pharmacological agents that have been studied and/or have demonstrated at least some promise for this purpose.

#### Anti-inflammatory and disease modifying agents

Considering the role that inflammation appears to play in both acute and chronic abdominal pain experience in IBD, in patients with suspected active disease, it is imperative to control the associated inflammatory process, as even milder forms of luminal inflammation may be associated with abdominal pain in IBD.^9,17,24^ An in-depth assessment of the influence that inflammation control plays in the natural history of IBD and patient prognosis is outside of the scope of this particular review. In general, however, any therapy that has the capability of positively influencing inflammatory activity in IBD also has the potential to positively impact abdominal pain in these conditions.

Importantly, many studies have relied upon clinical scores that incorporate an assessment of abdominal pain (eg, CDAI, HBI). In some cases, abdominal pain has been separately evaluated, however. For example, abdominal pain has been evaluated in the context of glucocorticoid therapy. One study evaluated 8 patients with established enteric/enterocolonic CD receiving 20–30 mg oral prednisolone daily for 6–9 weeks. Most of the patients reporting the presence of abdominal pain at the beginning of the study (5 of 7) described improvement in this symptom after receiving this treatment (based upon the abdominal pain assessment in the HBI).^152^ Notably, though, the mean differences in abdominal pain scores from the beginning to the end of this study were not significantly different. In another example, abdominal pain scores (rated on a patient-reported scale of 0–3) were significantly improved in UC patients receiving 8 weeks of upadacitinib when compared to individuals receiving placebo.^153^ Abdominal pain scores of CD patients receiving risankizumab were better than those reported by CD patients receiving placebo during the ADVANCE and MOTIVATE trials.^154^

It is also important to remember that certain IBD-directed therapies have been identified as potential causes of abdominal pain, including mesalamine,^155^ azathioprine,^156^ and vedolizumab (including when compared to individuals receiving placebo).^157,158^ Abdominal pain is also listed as a potential adverse effect for a variety of other IBD-directed therapies, including methotrexate, infliximab, adalimumab, certolizumab, golimumab, ustekinumab, and tofacitinib. Additionally, and in spite of the findings described above, glucocorticoids also have the potential to cause abdominal pain through a variety of direct and indirect mechanisms, including by contributing to the development of gastritis, peptic ulcer disease, adrenocortical insufficiency, pancreatitis, and even bowel wall perforation.

#### Analgesic medications

A wide variety of dedicated or incidentally analgesic agents have been used to manage abdominal pain in IBD. We summarize the studies specifically evaluating their use in IBD below.

##### Acetaminophen

Acetaminophen, or paracetamol, is one of the most commonly prescribed and/or self-administered analgesics for IBD.^159,160^ In fact, at least 1 study (involving a Swiss IBD cohort) suggested that it was the most commonly used pain medication in IBD.^161^ It is frequently a “first-line” analgesic, usually targeted to manage mild to moderate abdominal (or other) sources of pain, and previous clinical trials have suggested that acetaminophen is relatively safe in this setting.^54^ However, at least 1 meta-analysis demonstrated that acetaminophen use is associated with an increased risk of flare in CD.^162^ Further study is warranted to more carefully examine this relationship.

##### Nonsteroidal anti-inflammatory drugs

NSAIDs (including over-the-counter aspirin, ibuprofen, naproxen, and other prescription-strength varieties) are frequently used in the setting of IBD for extraintestinal and other sources of pain.^54^ However, as previously indicated, these medications have been associated with a variety of detrimental impacts to the gastrointestinal tract, including inflammation, ulceration, and bleeding and, as a result, IBD patients may actually experience abdominal pain when taking NSAID mediation.^53,54^ There is also evidence to suggest that NSAID use puts IBD patients at increased risk of developing flares of their disease.^53,54,163^ Of note, this effect is disputed by the findings of at least 1 systemic review and meta-analysis.^162^ This may have been, in part, due to the finding that selective COX-1 and COX-2 inhibitors have not been explicitly linked to IBD exacerbations.^54,164^ As a result, IBD providers and societal guidelines usually recommend avoiding the use of nonselective NSAIDs in this population, particularly on a chronic basis.^96,165^

##### Opioids

Opioid analgesics (including codeine, tramadol, hydrocodone, oxycodone, morphine, hydromorphone, and fentanyl) are used frequently in both the outpatient and inpatient setting to manage pain in IBD.^161,166^ Interestingly, studies directly evaluating the impact of opioids on pain, and abdominal pain in particular, have demonstrated no evidence that they help with this symptom.^17,167^ Additionally, these medications have been closely associated with a wide variety of adverse issues, including psychological and physical dependence, several gastrointestinal complications such as constipation, nausea, vomiting, and narcotic bowel syndrome.^55,56^ In IBD, they have also been specifically associated with other serious complications, including an increased risk of infections, worsening disease activity, poorer quality of life, and death.^168,169^ Considering these factors, opioid administration (particularly in nonoperative settings) is frequently contraindicated as a treatment for abdominal pain in the setting of IBD.^165,170^ If opioid administration is considered for management of chronic abdominal pain in this context, it is important to work with a pain specialist and/or the primary care provider/team to choose the safest option available, delineate clear guidelines and limitations for their use, and to optimize monitoring for abuse, adverse effects, and efficacy.^171^ Use of a multidisciplinary approach is particularly important in the setting of opioid dependence and/or opioid use disorders.^172^ If patients using opioid medications require de-escalation or discontinuation of these agents, providers should utilize established weaning protocols in order to minimize adverse symptoms and complications associated with opioid withdrawal.^173^

##### Antispasmodics

 These are a heterogeneous group of medications that impart effects through a variety of different mechanisms, including cholinergic receptor (eg, dicyclomine, hyoscyamine) and calcium channel inhibition (eg, alverine, otilinium, pinaverium). In part due to anecdotal reports of improved IBD-associated pain experience with their administration,^174^ antispasmodics have been used to target presumed gastrointestinal spasms or “hyperactive” motility. Several antispasmodics (including those listed above) have also demonstrated efficacy in improving abdominal pain scores of patients with brain–gut disorders, including IBS and functional dyspepsia.^175^ This is relevant to the care of IBD patients due to the array of potential contributors to altered gut motility in these conditions, including chronic inflammation, luminal narrowing, and even excess gas production. As these conditions are relatively common, including in patients with IBD, there are frequent opportunities to employ them to assist with abdominal pain management in this setting. However, no studies to date have demonstrated a specific benefit to abdominal pain experience in IBD. Importantly, these agents are also associated with problematic adverse effects including dry mouth, urine retention, blurred vision, tachycardia, and drowsiness.^176^

##### Anticonvulsants

 Anticonvulsant (or antiepileptic) medications, including gabapentin and pregabalin, have been studied as potential visceral analgesics as well. These are a diverse group of medications (also known as gabapentinoids) with varied mechanisms of action, including antagonism and/or modification in expression of voltage-gated calcium channels (VGCCs) and voltage-gated sodium channels (VGSCs).^177^ As with antispasmodics, they have demonstrated utility in managing abdominal pain in the setting of brain–gut disorders such as IBS. Pregabalin has been shown to increase the mean threshold for noxious sensation related to barostatic rectal distension in IBS patients and, in a randomized double-blinded placebo-controlled trial, has been demonstrated to improve abdominal pain scores in IBS patients.^178,179^ No studies to date have been undertaken to evaluate the analgesic efficacy of these agents specifically in IBD.

##### Antidepressants

There are numerous classes of antidepressant and anxiolytic medications that work through myriad mechanisms of action, including the selective serotonin reuptake inhibitors (SSRIs) (examples including fluoxetine, paroxetine, citalopram, escitalopram, sertraline), serotonin and norepinephrine reuptake inhibitors (SNRIs) (examples including venlafaxine, desvenlafaxine, duloxetine), and the TCAs (eg, amitriptyline, nortriptyline, desipramine, imipramine). There has been substantial interest in the potential of antidepressants as visceral analgesics for IBD. This is in part due to the demonstration in previous studies that TCAs improve abdominal pain in IBS.^130,180^ One small prospective study of patients diagnosed with IBD and IBS receiving any TCA found that a general symptom profile improved over time (though not necessarily abdominal pain).^181^ However, none of these agents have yet been studied directly to specifically assess their impact on abdominal pain control in IBD patients. Nonetheless, recent expert guidelines have suggested that practitioners should consider use of antidepressants in IBD patients experiencing “functional” abdominal pain.^170^

Antidepressants have also been studied as adjunctive therapies to help manage the inflammation associated with IBD itself, and the results have been mixed. A large population-based study conducted in Canada demonstrated that the development of incident depression was associated with a higher risk of both CD and UC, but this risk was reduced by use of SSRIs and TCAs in CD and SSRIs, SNRIs, serotonin modulators, and mirtazapine in UC.^182^ A separate population-based study performed in Denmark also demonstrated that IBD patients using antidepressants were less likely to exhibit disease activity, and this association was particularly strong for new antidepressant users.^183^ Case studies have described CD patients who went into disease remission after initiation of bupropion (a purported norepinephrine and dopamine reuptake inhibitor).^184^ In a small RCT involving 44 “IBD” patients on mesalazine, patients receiving concomitant duloxetine exhibited significantly better improvements in quality of life and severity of physical symptoms.^185^ However, in another RCT involving 26 CD patients in clinical remission, the SSRI fluoxetine was not superior to placebo in maintaining remission nor in its impact on quality of life.^186^ Additionally, in 1 recent population-based retrospective study, continuous use of SSRIs or TCAs was found to be associated with an increased risk of chronic corticosteroid use in IBD.^187^

##### Cannabis and cannabis derivatives

 As we have reviewed elsewhere,^188^ cannabis and its derivatives impart their effects through interactions with endocannabinoid [cannabinoid receptor type 1 (CB_1_R) and cannabinoid receptor type 2 (CB_2_R)] and nonendocannabinoid (eg, TRPV1, PPAR-alpha, PPAR-gamma) receptors found throughout the body. These agents have been implicated as safer analgesic options for many different conditions, though there is still a relative paucity of information about their efficacy and safety in this context.^188^ Notably, significant concern has been raised regarding the potential impact they have on brain development and function, including in young adults.^189,190^ This is also relevant because cannabis and cannabinoid use is common in IBD and is frequently used by patients for abdominal pain relief.^191–194^ Use of cannabis and its derivatives has been associated with improved abdominal pain experience (including in placebo-controlled studies) and reduction in the use of other analgesics (including opioids) in IBD.^195–199^

There has been long-standing interest in the anti-inflammatory potential of cannabis in IBD. An RCT trial evaluating low-dose cannabidiol in CD demonstrated no significant impact on disease activity.^200^ A follow-up RCT in mild to moderate UC using a higher dose of cannabidiol oil demonstrated symptomatic improvement in the treatment group (including reduction of abdominal pain).^201^ However, there was still no significant effect on inflammatory activity (and, notably, fewer patients were able to tolerate the increased dose). Perhaps most strikingly, in a phase 2 clinical trial, CD patients receiving the selective CB_2_ agonist olorinab demonstrated significant improvement in abdominal pain scores compared to control subjects.^106,202^

##### Antibiotics

This class of medications have demonstrated potential efficacy to address inflammation related to CD as well as in treating common complications associated with IBD, including infections and abscesses.^203^ As described previously, each of these phenomena may be associated with abdominal pain in IBD and so successful treatment of these issues may result in significant improvement in this symptom.

##### Analgesic agents currently used to manage visceral hypersensitivity disorders

 Several other medications have potential for analgesic effect in IBD, particularly in the setting of a concomitant visceral hypersensitivity disorder, such as IBS. These agents have not been studied rigorously in the context of IBD, though. *Opioid receptor agents*—Eluxadoline is a mu- and kappa-opioid receptor agonist and delta-opioid receptor antagonist that has demonstrated efficacy in managing bowel habit changes and abdominal pain in diarrhea-predominant IBS.^204^*Serotonin receptor modulators—*Several serotonin receptor modulating agents also have the potential to help, though they have not been rigorously studied specifically in human forms of IBD. These include alosetron, a selective serotonin receptor 3 (5-HT_3_R) antagonist that has previously been approved for the treatment of IBS-D (though carries a black box warning of ischemic colitis).^205,206^ Tegaserod, a selective serotonin receptor 4 (5-HT_4_R) agonist, has demonstrated efficacy in improving symptoms in IBS-C, including abdominal pain (though it had been temporarily withdrawn from the US market out of concern for potential adverse cardiovascular effects).^207^ Though constipation is less common in IBD populations, there is still analgesic potential in this setting. *Guanylate cyclase agonists—*Linaclotide (a guanylate cyclase C (GC-C) agonist) has demonstrated the capability of reducing abdominal pain in IBS-C patients, potentially through direct interaction with visceral nociceptors to reduce transmission of signals that can be interpreted as pain.^208^ Plecanatide is another GC-C agonist that is approved to treat both IBS-C and chronic idiopathic constipation,^209^ and that has also demonstrated efficacy in improving abdominal pain in these conditions.^210^*Calcium channel antagonists—*Lubiprostone (a stimulator of type 2 chloride channels in gut epithelia) has also been shown to specifically improve abdominal pain in IBS-C patients.^211^ Of note, peppermint oil (which includes several potential active constituents including L-menthol, which is purported to block calcium channels in smooth muscle^212^) has also demonstrated an ability to significantly improve IBS-associated symptoms and abdominal pain in particular,^212^ and has been used to manage abdominal pain in IBD patients,^213^ though no controlled studies in this population have been undertaken to evaluate this therapy thus far. *Sodium–hydrogen exchange inhibitor*—Tenapanor inhibits the sodium–hydrogen exchanger 3 (NHE3) antiporter in gastrointestinal epithelium. It has also been demonstrated to significantly improve abdominal pain in IBS-C patients.^214^ As with the preceding agents, tenapanor has not been specifically studied in the context of IBD.

## Management Strategies in the Clinic

While we have learned a great deal about abdominal pain in IBD thus far, there are still many holes in our understanding and challenges we face in regard to truly optimizing our management of this symptom. Considering this, it is essential for IBD providers to recognize our current limitations in knowledge and management of abdominal pain in this setting, in order to provide the safest and most effective strategies. In addition to the appropriately targeted use of the agents and technologies described above, there are relatively simple organizational approaches that are important to incorporate in this setting.

### Patient and Provider Education

The first, simplest, and possibly most important strategy, relates to patient education. It is of paramount importance that IBD providers clearly and effectively communicate what they understand about each patient’s condition and the causes of their symptoms, including source(s) of pain. Abdominal pain is one of the most common reasons that IBD patients (and individuals with digestive disorders in general) seek out medical care. Pain is commonly a frightening, disruptive symptom that leads to significant anxiety and reduced quality of life, in part because patients do not always recognize what is driving it or how best to take care of it. IBD providers are critically important for helping patients understand the processes responsible for this symptom as well as the diagnostic and therapeutic approaches currently available that are best suited to address their needs, while avoiding therapies and behaviors that may do more harm (eg, NSAIDs, opioids). It is, therefore, essential for IBD providers to receive appropriate training throughout their careers on how to properly identify and manage abdominal pain in these conditions. It is also helpful to implement dedicated protocols (in both the outpatient and inpatient settings) with clear care guidelines in order to streamline the identification of abdominal pain sources, eliminate more common risk factors for its development, and to avoid potentially toxic analgesic agents (including opioid administration).^215,216^ It may also be beneficial to incorporate these protocols into the electronic medical record to help automate the protocol and deliver appropriate prompts to providers and/or their patients when higher risk behaviors or therapies are identified (eg, tobacco use, opioid administration, etc.).^217,218^

### IBD Centers and Specialty Clinics

Considering the many and varied challenges most healthcare providers deal with on a daily basis, keeping up with best practices in regard to the management of abdominal pain in IBD may not be possible for everyone. In this setting, if providers are not comfortable managing IBD patients or their symptoms, it is just as important then to refer patients to a dedicated provider and/or center with expertise in these conditions. It is also essential to refer to these specialists as early as possible in the disease course. The physical and organizational care setting may also make a big difference in this regard. Dedicated IBD centers of care are helpful for providing tailored management of these patients, as they tend to concentrate the relevant expertise and resources necessary for their care.^219^ These centers are also frequently designed to provide multidisciplinary approaches for both inpatient and outpatient care,^215^ facilitating concurrent management by several specialties (eg, gastroenterology, colorectal/gastrointestinal surgery, nutrition, psychiatry, pain management, etc.) while allowing for relatively expedited evaluations and recommendations for each patient, as well as patient-centered conferences and educational forums.^220^ There is evidence that when specialty clinics (eg, nutrition, psychiatry) are incorporated into the framework of IBD centers, patient outcomes related to abdominal pain experience are improved.^221,222^ Notably, the care at these centers is frequently facilitated by nurse navigators. Their participation alone can also result in measurable impacts on patient satisfaction and pain-related factors.^223^

## Future Directions

We have learned a great deal about abdominal pain, including its drivers in IBD. Unfortunately, gaps remain in our understanding of the pathophysiology underlying abdominal pain in this setting, and there is still much to be learned about how best to use current treatment options, as well as analgesics currently under development. These limitations are clearly impairing our ability to optimally care for millions of people who suffer with abdominal pain on a daily basis. IBD patients and providers need novel, more effective, safer methods for the detection, characterization, and treatment of abdominal pain. While no clear solution exists for any of these problems, there are promising areas of current investigation in each regard.

### Larger and More Refined Studies of Abdominal Pain in IBD

Investigators need to find more effective methods for studying abdominal pain, particularly in the setting of IBD. In recent years, laudable efforts have been made to bring experts together to specifically strategize on how to better study and manage this problem.^224^ There are also multicenter data registries and biobanks as well as national and international consortia in development that have the potential to effectively study larger numbers and more comprehensive forms of relevant patient data as well as biological specimens.^225,226^ These efforts provide hope that future studies generated by these resources will provide more refined insights into the nature of abdominal pain in IBD.

However, in addition to evaluating larger and better described patient cohorts paired with biological samples, it is clear that we also need to further refine our modeling assumptions about abdominal pain in IBD. There has been a tendency to try to extrapolate lessons learned about abdominal pain in other conditions (including IBS) in order to make inferences about what drives this symptom in IBD. While there is potential pathophysiological overlap between certain forms of IBD and IBS (as well as other digestive disorders), both of these conditions represent several heterogeneous disorders, each of which can vary substantially in regard to symptom derivation. The IBDs themselves are each associated with many unique factors (including inflammatory and anatomical considerations) that are not necessarily relevant to any other conditions (as phenotypically similar as they may appear to be). In future studies of abdominal pain in IBD, in addition to carefully phenotyping their IBD study participants, investigators should consider concurrent study of other conditions associated with abdominal pain to compare their symptomatic and biological features and to help delineate key pathophysiological traits that differentiate them.

### Understudied Topics and New Study Models

There is also need for more careful study of relatively neglected issues to date. This includes more systematic assessment of traits and exposures known to influence pain experience, such as age,^227^ sex,^228^ stage of the menstrual cycle,^229^ diet,^111^ and medication usage. This also includes further study of poorly understood phenomenon, such as the impact of abdominal wall pain in this population (a topic that is particularly relevant considering the number of IBD patients who undergo abdominal surgery during their lifetime). In spite of the fact that there are clinical methods to differentiate this cohort of patients and a variety of potential therapeutic approaches, there is a relative paucity of studies describing this issue in IBD. There are also a variety of heretofore under-evaluated potential therapeutic options waiting to be more carefully studied. As described above, many of these approaches have already demonstrated promise for addressing abdominal pain in IBD and other conditions associated with chronic abdominal pain. This list includes cannabis derivatives, psychological and behavioral therapies, brain and nerve stimulation treatments, lifestyle modifications (including those related to diet and exercise), and the more novel pharmacologic options described above.

The use of new and relatively unconventional models to study abdominal pain could also be useful in this context. One such example involves silent, or hypoalgesic, IBD. This is a striking, and surprisingly common, condition in which certain IBD patients simply do not perceive nociceptive (or pain-inducing) stimulation during periods of active disease (unlike most other people). A clearer understanding of what keeps silent IBD patients from perceiving abdominal pain could lead to better targeted, safer, and more effective visceral analgesic therapies as well as more objective methods for assessing patient pain experience. For example, we recently demonstrated that silent IBD patients were more likely to be homozygous for a polymorphism associated with a voltage-gated sodium channel (NaV1.8) gene (SCN10A).^230^ Targeted study of novel mechanisms such as these could provide insights leading to more effective and safer analgesic options in the future.

### Updated Methods to Study Abdominal Pain

Simply identifying and measuring pain of any type has been a tremendous challenge. Part of the issue lies with the fact that investigators have utilized widely varying measures and descriptors of abdominal pain in their studies that can be difficult to compare to one another. This is in part due to the fact that while there are many survey tools available to evaluate abdominal pain, none have been specifically designed or validated to be used in the context of IBD. Studies are clearly needed to validate and standardize methods for abdominal pain characterization in IBD.

Presently, there are also no tests that can objectively assess a patient’s abdominal pain experience and so providers must rely on inconsistent and/or inaccurate means for assessing patient pain intensity and medication requirements.^3,231^ For years, investigators have been searching for reliable methods to adequately screen for and quantify patient pain experience. As described above, investigators have used barostatic rectal distension to assess abdominal pain experience in several conditions. However, there are several practical limits to use of this technique in the setting of IBD, particularly during active phases of the disease. There may be less invasive testing options, though. One more recent example is the use of skin conductance testing (SCT). SCT is performed by applying a constant, low voltage to the skin between 2 or more electrodes. During activation of the sympathetic nervous system (which frequently occurs in the setting of pain), humans perspire and this increases electrical conductance, thus providing a potential objective, biophysical measure of pain experience.^232^ This approach, and variations of it such as the “normalized” SCT,^233^ has demonstrated potential utility for identifying and characterizing somatosensory pain in several settings, including during sedated procedures to help anesthesiologists determine how and when to administer analgesic medication.^232,234^ It is possible that this method could be used to better screen for and characterize abdominal pain in individuals with IBD. Further study on these, and other techniques designed to evaluate potential biomarkers of pain, is necessary. If successful, these methods could revolutionize the manner in which providers approach pain management.

## Summary

**Figure 3.**
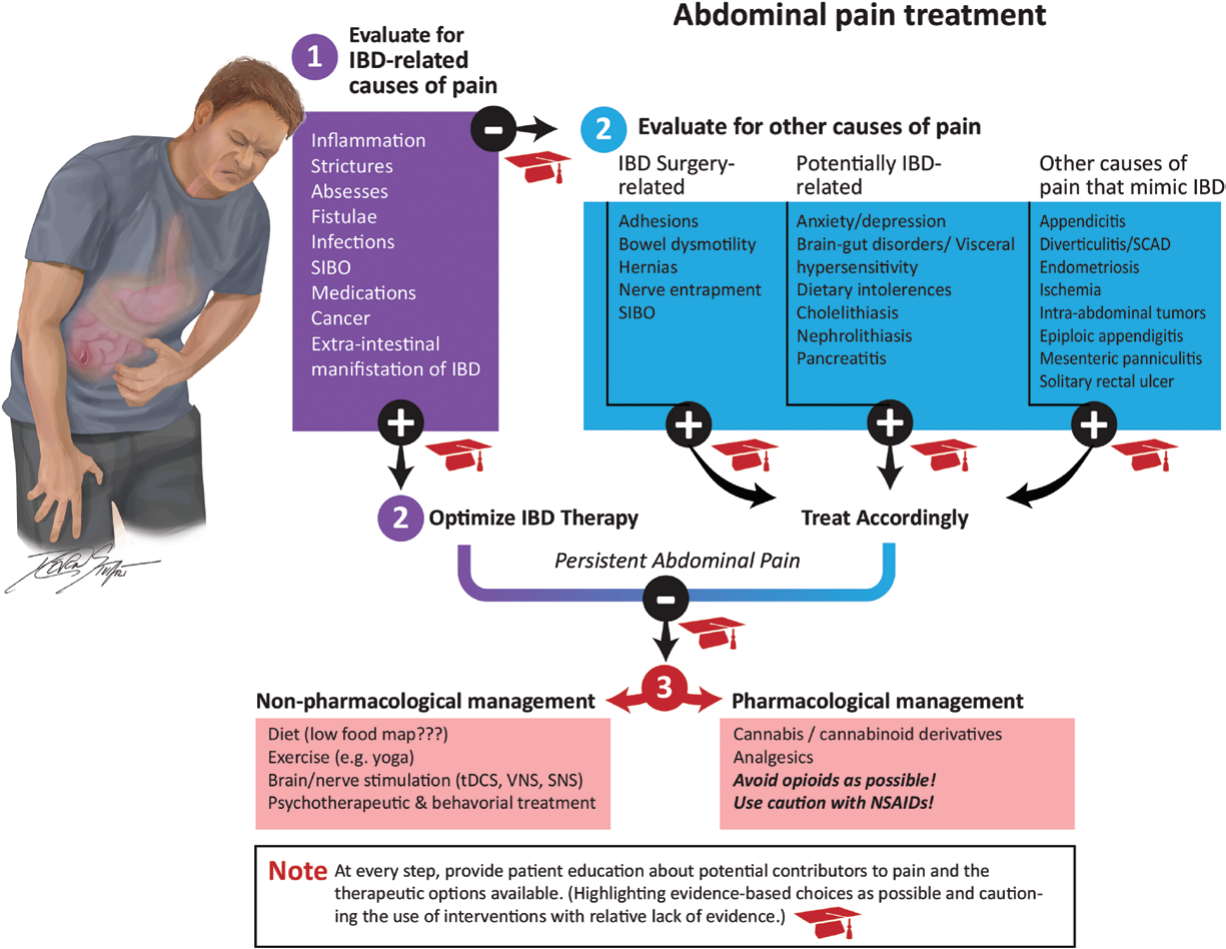
Proposed approach to the management of abdominal pain in inflammatory bowel disease.

In summary, abdominal pain is a very common, critically important and still poorly understood symptom in IBD that is associated with significant consequences to the patient, provider, and society at large. It is frequently time- and cost-intensive to manage, current tests are limited in their ability to consistently identify the cause(s) of this symptom, and available analgesics are frequently toxic and/or ineffective in this context. There are a variety of promising methods and technologies currently being studied to manage abdominal pain in IBD, but we still have a great deal to learn about visceral pain pathophysiology and treatment. Moving forward, investigators who manage and study this issue will need to rely on more sophisticated, comprehensive, and integrated methods to characterize their patient populations and to find more effective, less problematic treatment options. It is becoming increasingly obvious that, as with most aspects of IBD care, a multidisciplinary approach is essential in this regard as well. Finally, patients must be an integral part of the decision-making process in relation to managing symptoms such as abdominal pain. Not only are they best suited to recognize and characterize their own abdominal pain experience, but patients are the only ones that can truly assess the efficacy of each particular treatment. Using the information outlined above, we have included a proposed approach to managing abdominal pain in IBD patients (Figure 3). Ultimately, regardless of the causes or the treatment choices, safe and effective abdominal pain management in IBD requires a carefully integrated, patient-centered approach.

## Data Availability

Data sharing not applicable—no new data generated.
